# Severe haemophilia a in a preterm girl with turner syndrome - a case report from the prenatal period to early infancy (part I)

**DOI:** 10.1186/s13052-020-00892-7

**Published:** 2020-09-07

**Authors:** Agnieszka Berendt, Monika Wójtowicz-Marzec, Barbara Wysokińska, Anna Kwaśniewska

**Affiliations:** 1 Department of Obstetrics and Pathology of Pregnancy, Medical University of Lublin, Staszica 16, 20-081 Lublin, Poland; 2 Department of Paediatric Cardiology, Medical University of Lublin, Prof. A. Gębali 6, 20-093 Lublin, Poland

**Keywords:** Haemophilia, Turner syndrome, Hemorrhage, Cerebral intraventricular hemorrhage, Infant premature, Infant newborn, Female, Genetic diseases, inborn, Blood coagulation disorders, inherited, Gonadal dysgenesis

## Abstract

**Background:**

Bleedings are more frequent in the population of preterm children than among those born at term, much less in older children. The reasons for such bleedings in preterms include plasma factor deficiencies, immaturity of small vessels in the germinal matrix region, prenatal hypoxia or sepsis. They affect the brain tissue, the gastrointestinal tract and the respiratory system, or are manifested by prolonged bleedings from injection sites. Haemophilia is a rare cause of haemorrhages in the neonatal period, and in the female population it is even seen as an extremely rare disorder. Its aetiology in girls is diverse: inheriting defective genes from their parents, skewed X inactivation or a single X chromosome.

**Case presentation:**

The article presents a case of a preterm girl born in the 28th week of pregnancy, who was diagnosed with severe haemophilia A stemming from the absence of the X chromosome. The girl’s father is healthy, but her mother’s brother suffers from haemophilia.

On the second day of the child’s life, a prolonged bleeding from the injection site was observed. A coagulation profile revealed prolonged APTT which pointed to haemophilia A diagnosis. Moreover, a marked clinical dysmorphy, female sex and a negative family history on the father’s side led the treating team to extend the diagnostic procedures to encompass karyotype evaluation. The girl was diagnosed with Turner syndrome. No bleeding to the central nervous system was observed during her hospital stay.

**Conclusions:**

Preterm children belong to the risk group of bleeding into the central nervous system or haemorrhages in the course of sepsis. Rare causes of such bleedings should also be borne in mind, including haemophilia.

The initial symptoms of haemophilia in preterm children occur in the first days of their lives, which is connected with a number of invasive procedures required in that period.

Genetic conditions may coexist with one another. Arriving at one diagnosis does not mean one should abandon further diagnostic procedures in cases where additional atypical symptoms are present which do not match the clinical image of a primary disease.

## Background

Haemophilia is an X-linked, recessive genetic condition. The defect regards the F8 gene product – coagulation factor VIII, the lack of which leads to coagulation disorders. Haemophilia is considered a rare disease. According to Orphanet (the portal for rare diseases and orphan drugs), the prevalence of haemophilia A is 1–9/100000 [1]. The possible causes of haemophilia in females include homozygosity of the haemophilia gene (i.e. when the child’s father has the disease and her mother is a carrier), extreme X-chromosome inactivation (skewed X inactivation) in a heterozygote (despite heterozygosity, the girl presents with phenotypic features of haemophilia), a single X chromosome (Turner syndrome), and de novo heterozygotic mutations. The presented case involves an early manifestation of haemophilia in a girl born in the 28th week of pregnancy in whom, in the course of a further diagnostics, haemophilia was found to be secondary to Turner syndrome (TS). The coincidence of those two units is extremely rare. Only eight cases have been described to date [2–9].

It is worth to emphasise that haemophilia in female newborns as a cause of haemorrhages is extremely rare disorder. Therefore, detailed diagnosis should be performed and second coexisting disease should be taken into account.

## Case presentation

### Family history

The patient’s mother was 30 at the time of labour, and it was her second pregnancy. The first pregnancy was terminated by caesarean section due to macrosomia of the male foetus with birth weight of 4750 g. Following the caesarean section, in the postpartum period, the mother had an episode of excessive vaginal bleeding. She described the state of health of her first child as good.

The family history includes severe haemophilia A in the mother’s brother. The child’s father is healthy. The mother’s brother did not present with any clinical symptoms of his disease in the neonatal period. Haemophilia signs appeared as late as at the age of 3, when he experienced heavy bruising and bleeding to joints and muscles. He was then diagnosed with severe haemophilia A with factor VIII at 0.1%. Apart from the brother with haemophilia A, the mother did not report any close relatives who would display signs of suspicious bleedings, haemorrhages after medical procedures or death at a young age.

### Prenatal period

**Fig. 1:**
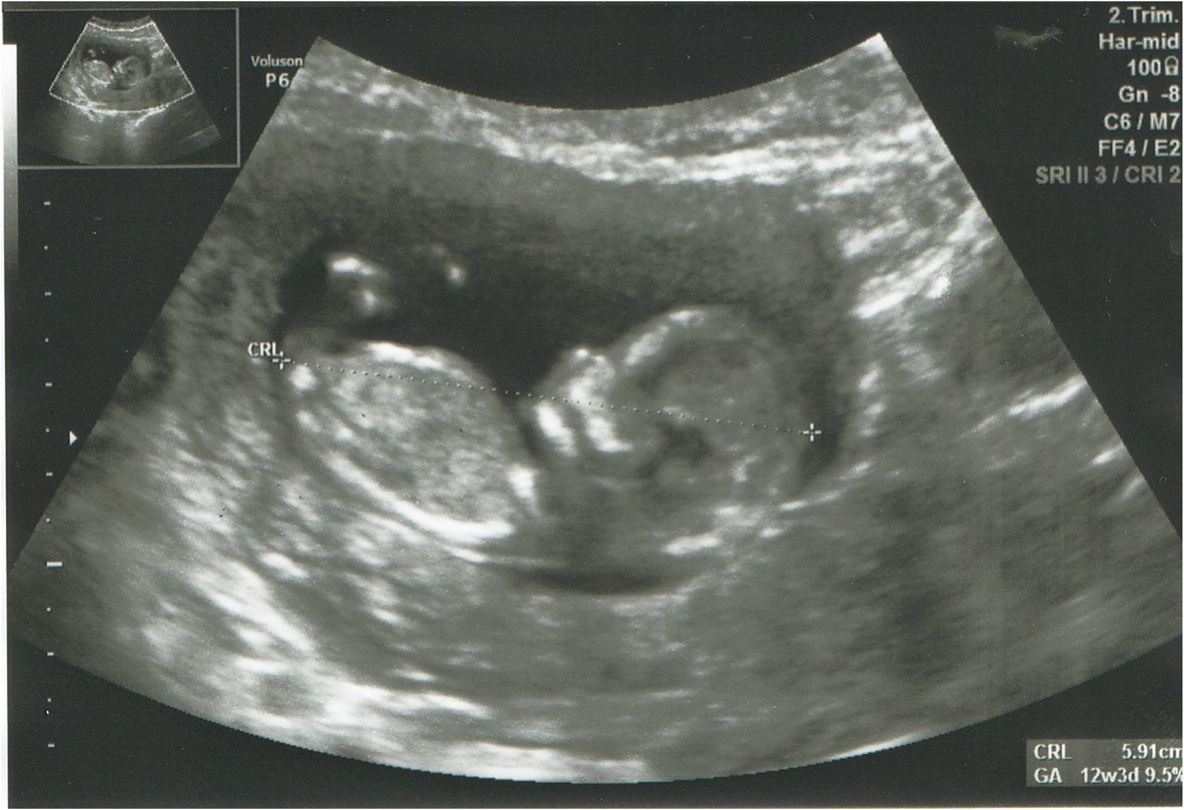
Fetus profile in 12th week of pregnancy (NT- 1.2 mm and CRL at 5.9 mm). (written consent to publish was obtained from the patient’s parents)

**Fig. 2:**
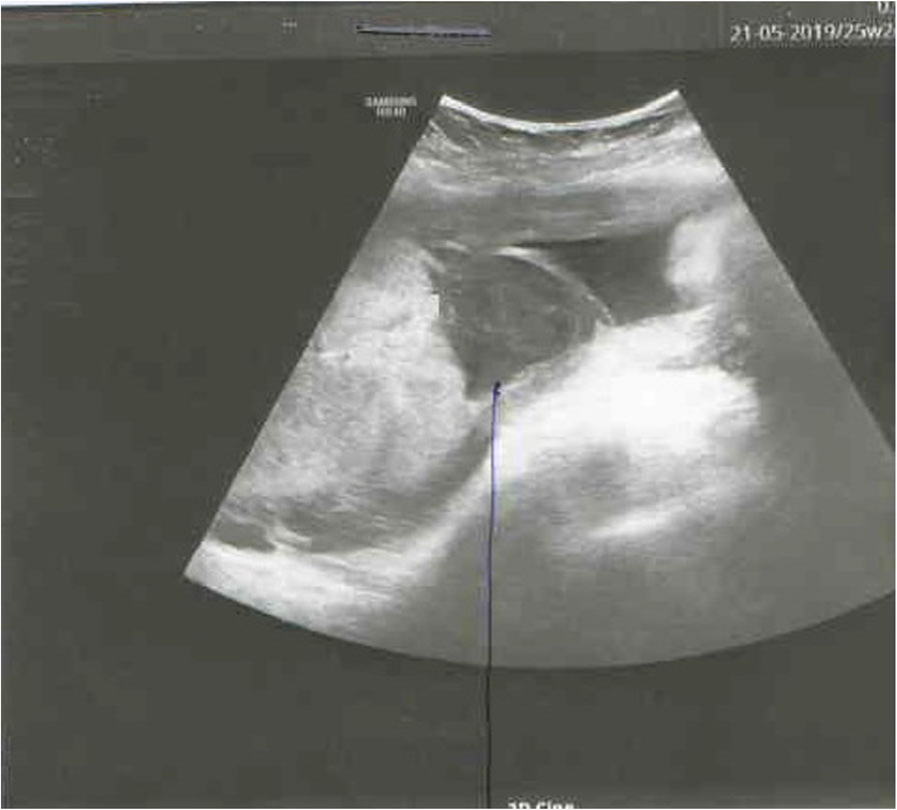
A retroplacental hematoma (written consent to publish was obtained from the patient’s parents)

A prenatal ultrasound examination performed in the 12th week of pregnancy showed a foetus with normal biometrics, a visible nasal bone, nuchal translucency (NT) of 1.2 mm and CRL at 5.9 mm [Fig. 1]. The subsequent ultrasound examination performed in the 20th week of pregnancy also pointed to normal foetal biometrics, with an estimated body weight of 350 g. In the 25th week of pregnancy, the mother attended the hospital due to vaginal bleeding [Fig. 2]. The foetus was then described as hypotrophic with an estimated body weight of 741 g, a normal profile, an image of 4 heart cavities, three mediastinal vessels, and foetal and extrafoetal flows. The mother was given a full course of steroid therapy. The bleeding stopped. In the 28th week, the patient again reported to the district hospital due to vaginal bleeding. She was again put on a full cycle of steroids and foetal neuroprotection with magnesium sulphate. The mother’s coagulation profile showed no deviations from the normal range. Due to signs of premature placental detachment and silent oscillation of the foetus, the pregnancy was terminated through caesarean section in its 28th week. The histopathological examination of the placenta showed an eccentric attachment of the umbilical cord with an unchanged foetal surface. The placenta was, however, changed on the mother’s side and showed a grey lesion following a retroplacental hematoma with a diameter of 70 mm; the two-vessel umbilical cord with the coiling index of 0.23.

### Neonatal period

The premature girl was delivered through caesarean section with extremely low birth weight of 880 g. She was evaluated for 6–6–7-7 points in the Apgar score. After birth, the preterm infant required lung expansion followed by mechanical ventilation. The girl received intratracheal Poractant alfa and vitamin K intramuscularly. No bleeding was observed.

**Fig. 3:**
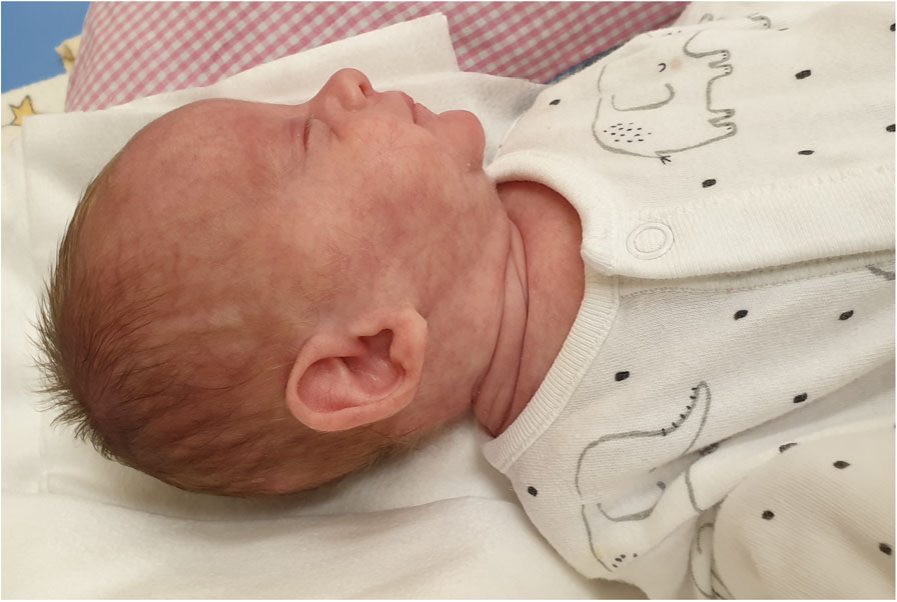
Face profile in 2nd month of life: big, low-set ears rotated to the back, micrognathism (written consent to publish was obtained from the patient’s parents)

**Fig. 4:**
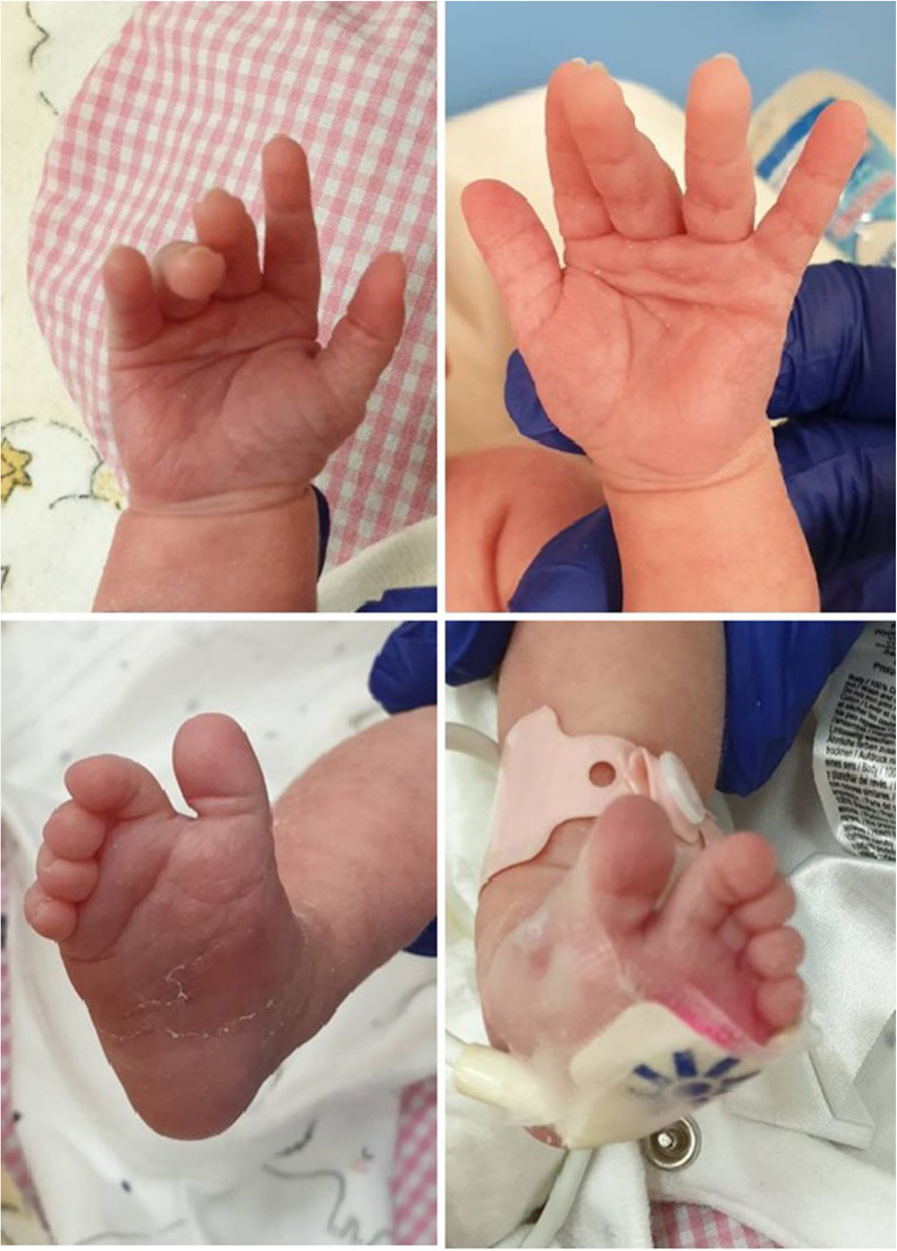
A single transverse palmar crease. A single crease on the feet, a sandal gap. (written consent to publish was obtained from the patient’s parents)

**Fig. 5:**
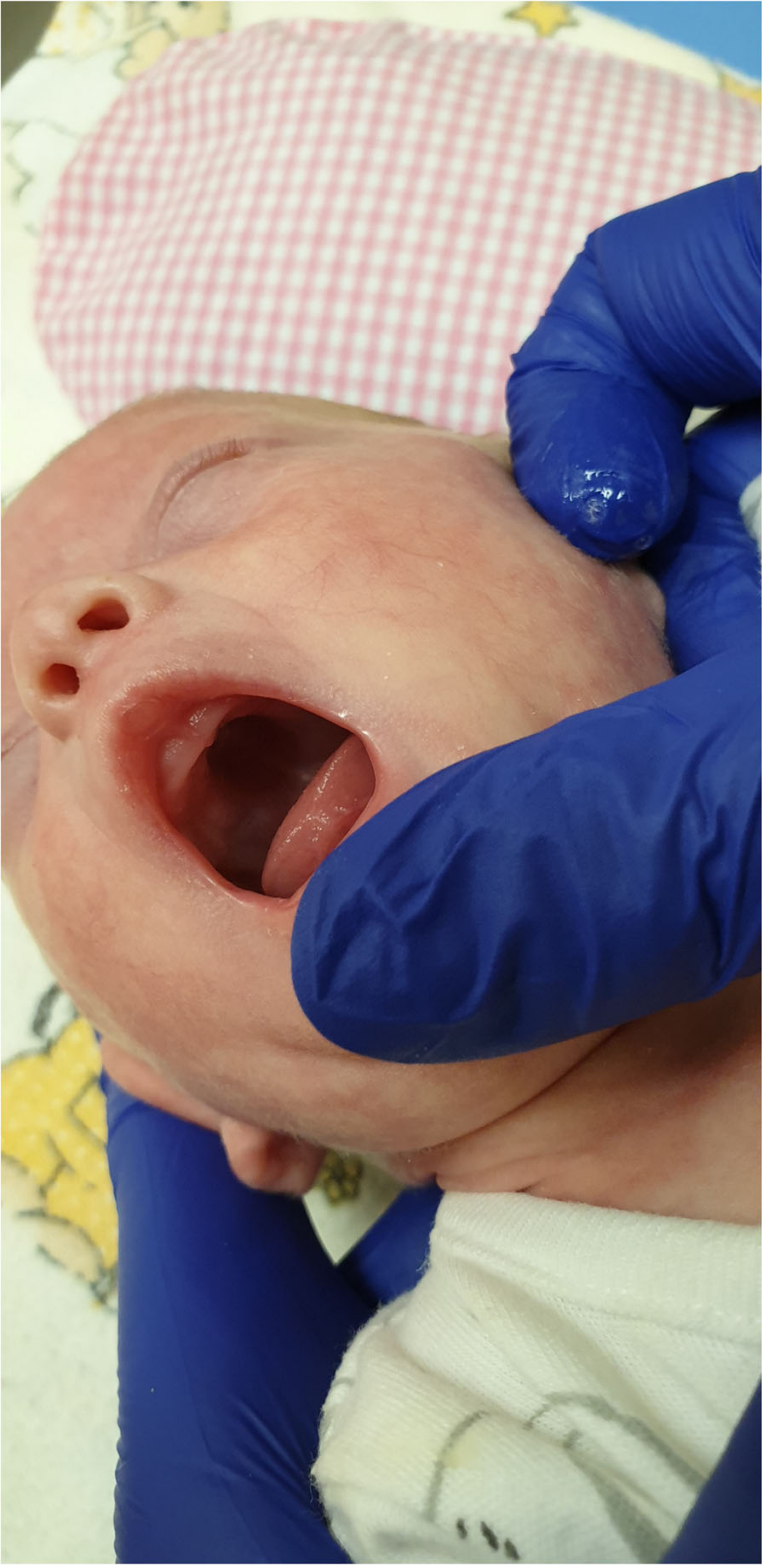
A gothic palate (written consent to publish was obtained from the patient’s parents)

**Fig. 6:**
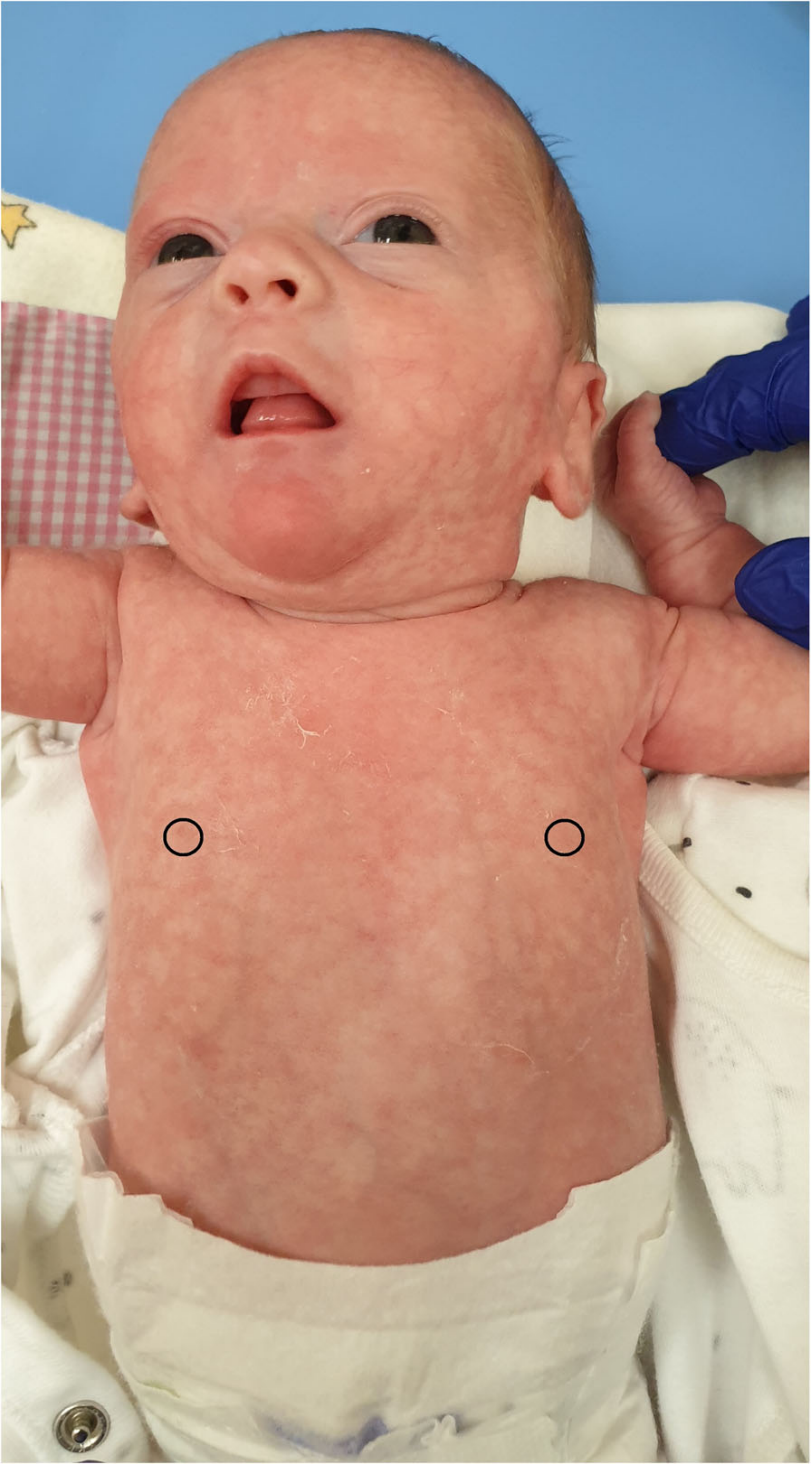
Widely spaced nipples, livedo reticularis (written consent to publish was obtained from the patient’s parents)

Apart from features of extreme prematurity, the physical examination showed signs of dysmorphy, i.e. big, low-set ears rotated to the back, micrognathism, a gothic palate, a single transverse palmar crease, a single crease on the feet, a sandal gap and widely spaced nipples (Figs. 3, 4, 5 and 6).

At the 36th hour of her life, the girl experienced bleeding from the site of sample collection for laboratory tests. The repeated administration of vitamin K and etamsylate were ineffective. The bleeding was managed only after the introduction of fresh frozen plasma (FFP). The tests showed indeterminable APTT with normal PT. Due to suspected haemophilia, the levels of coagulation factors were checked. The factor VIII level was found to be low at 0.1%, with normal values of factors IX, XI, XII and von Willebrand factor (vWF). The girl was diagnosed with haemophilia A. Circulating anticoagulant (anti-Factor VIII (FVIII) antibodies) was tested in the first days of the girl’s life, yielding a negative result. During her hospital stay, the patient had numerous infusions of factor VIII coagulation: because of the bleeding from the injection site, before and after the retinal laser photocoagulation procedure due to retinopathy of prematurity. The level of factor VIII (anti-Factor VIII (FVIII) antibodies) was monitored. It was found that its level was higher in the subsequent tests. Following other bleeding episodes, the girl was treated with recombinant factor VIIa (rFVIIa).

The patient was subjected to regular ultrasound check-ups for any potential intraventricular bleeding. The cerebral image was normal throughout the entire hospitalization.

In the 3rd week of the girl’s life, a nodule appeared on her head, which was oozing blood. The bleeding stopped only after the administration of factor VIII.

The haemophilia diagnosis, no signs of the disease in the mother, female sex and dysmorphic features were indications for further diagnostics and karyotyping. The examination showed two cell lines 45X, 45X + mar, which confirmed Turner syndrome. The presence of the SRY gene was ruled out.

**Fig. 7:**
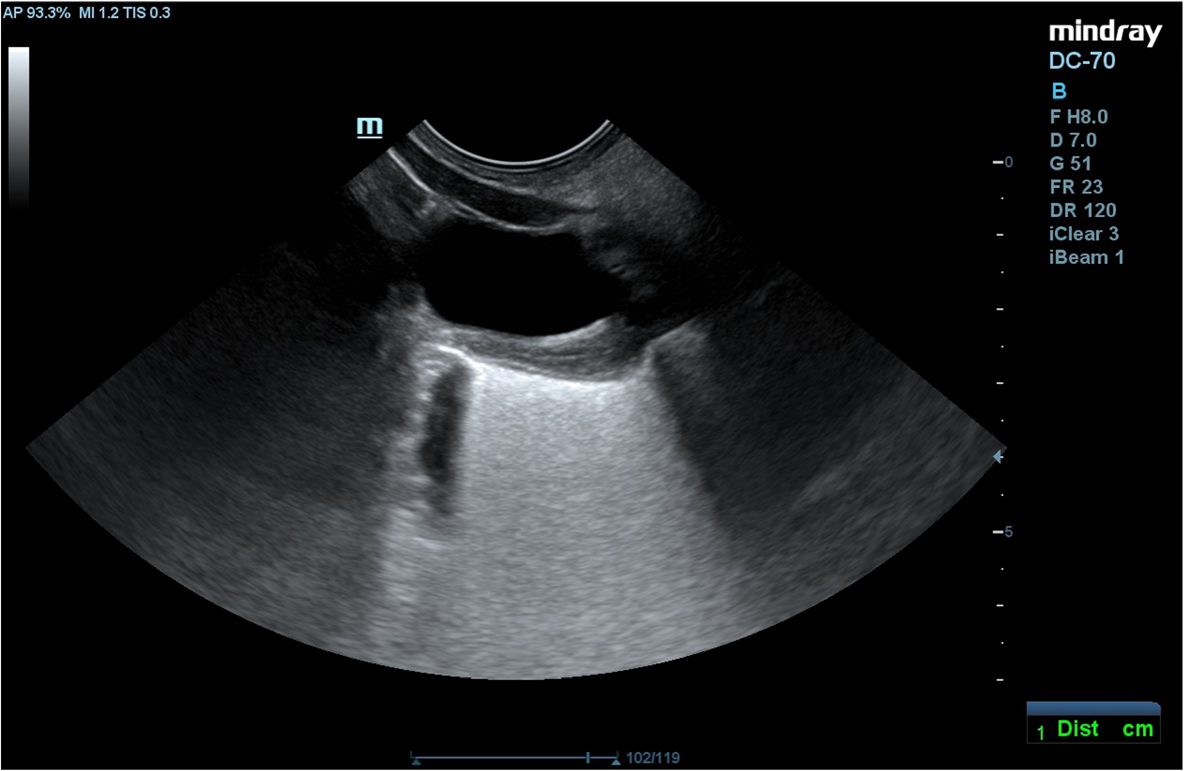
An uterus with a hyperechogenic endometrium; ovaries could not be seen (written consent to publish was obtained from the patient’s parents)

The diagnosis of Turner syndrome suggests a further evaluation for other congenital defects, including defects of the circulatory system. An abdominal ultrasound examination did not reveal any abnormalities in the urinary tract structure. The uterus was found with a hyperechogenic endometrium; ovaries could not be seen (Fig. 7).

The echocardiography revealed an atypical shape of the aortic arch, which was difficult to see in a 2D view; 2 vessels originating from the aortic arch at various angles were found. The tricuspid aortic valve had a normal morphology and size. The Holter ECG examination did not record any significant heart rhythm disorders. The girl remains under constant care of a haematologist, an endocrinologist and a cardiologist. Such aspects as education and psychological care of the parents should also be emphasised.

## Discussion and conclusions

The presented case shows an early manifestation of severe haemophilia A in a girl in whom the disease was also found to be secondary to Turner syndrome. The coincidence of these conditions is extremely rare. The available literature in the PubMed database cites 8 cases of haemophilia and Turner syndrome coexisting in the paediatric population [2–9]. In most cases, it is severe haemophilia. The diagnosis was made following the first clinical symptoms which were typical of this disease: frequent bruising, recurrent bleeding after surgeries and spontaneous bleeding to joints after small injuries.

In the case of severe haemophilia, the diagnosis is made in the first 2 years of the child’s life, and in moderate haemophilia – up to the age of 5.

The available literature points to the fact that the Turner syndrome diagnosis is usually incidental, with genetic tests revealing aneuploidy of the X chromosome. The authors do not elaborate on the potential dysmorphy or clinical features of the syndrome. A few of them mention short stature in later years.

This paper describes the ninth case of haemophilia and Turner syndrome coincidence. Both diagnoses were made in the neonatal period based on the following clinical symptoms: a prolonged bleeding from the injection site, a positive family history and marked dysmorphic features of the child. At the same time, the presented case report describes the first dual diagnosis in a preterm child.

The PubMed database contains descriptions of 9 cases of haemophilia A found in preterm children [10–17]^.^ The patients’ age was between the 25th and 33rd week of pregnancy. In the majority of these cases, the diagnosis was made on the first day of the child’s life due to a positive family history of the condition. The remaining cases saw the diagnosis on the second day, after an episode of a prolonged bleeding from the injection site, which was the first symptom of the disease. In one case, the diagnosis was incidentally made during routine laboratory tests before an elective repair of inguinal herniation. All presented cases were male preterm children. Apart from the case described here, no other similar example of extreme prematurity in a female child with coexisting haemophilia and Turner syndrome has been found (Table 1).

**Table 1:** Synopsis of reported cases of preterm infants with diagnosis of haemophilia

No	GA	Mother carrier	Sex	Diagnosis	IVH	Publication	Cause of diagnosis
1	28	yes	male	4 h	I	Gale 1998	family history
2	28	yes	male	at birth	I	Bidlingmaier 2004	family history
3	30	yes	male	at birth	no	Bidlingmaier 2004	family history
4	33	yes	male	at birth	no	Kraft 2008	family history
5	33	yes	male	at birth	I	Kraft 2008	family history
6	26	no	male	6 h	no	Gelbart 2009	prolonged bleeding from a venipuncture site
7	25	yes	male	at birth	I	Cartledge 2011	family history
8	26	no	male	87 day	no	Fink 2013	accidentally before operation of hernias
9	33	no	male	2 day	I	Kooiman 2015	prolonged bleeding from venipuncture sites
10	28	yes	female	10 day	no	This article	prolonged bleeding from a venipuncture site

*No* numero, *GA* gestational age, *IVH* intraventricular hemorrhage.

Having in mind that: the girl has haemophilia, her mother’s brother was also diagnosed with this condition and the child’s father is healthy, it could be concluded that the girl’s mother was the carrier of a defective haemophilia gene. She had not been subjected to any factor VIII level tests or genetic examinations.

As mother-carriers usually display normal or lower-limit levels of factor VIII, the test is not used to identify carriers. They are found in the population only with the aid of genetic testing [18, 19].

Carriers do not present with symptoms typical of haemophilia; however, diminished levels of VIII coagulation factor might result in easily bruising, tendency to massive bleeding after surgeries, heavy menstrual bleedings or bleeding during ovulation. What is more bleeding after giving birth may occur, as in the presented case. It occurs because the levels of factor VIII increase significantly in pregnancy, which reduces the risk of bleeding in carriers of haemophilia A. However, the high levels of factor VIII during pregnancy fall back to lower levels after delivery [18, 19] resulting in serious bleeding.

Most carriers have normal pregnancies without any bleeding complications. There does not appear to be a higher risk of miscarriage in carriers of haemophilia. However, the analysis of 149 pregnancies shows haemophilia carriers displaying the rations of the premature deliveries (8.2%). The ration of the bleeding complications during pregnancy was 18.7%, in 2.7% of the cases transfusion was necessary [20].

The mothers with a positive haemophilia history can benefit from prenatal diagnostics, i.e. chorionic villus sampling (CVS) or amniocentesis for determining whether the defective genes were passed on to the child. Also, assisted reproduction methods help to avoid the risk connected with transmitting the condition to the children.

Signs of an abnormal bleeding tendency in the neonatal period, which are characteristic of haemophilia, include a prolonged bleeding from wounds which lasts for days or even weeks, e.g. a prolonged bleeding from puncture sites, bruising after minor injuries, bleeding following minor injuries which can occur even as late as few days after the said injuries, or repeated bleeding from wounds. Spontaneous bleedings in the central nervous system CNS (subgaleal haemorrhage, intracranial haemorrhage), adrenal glands or the gastrointestinal tract may also occur. In early childhood, such haemorrhages involve mainly joints and muscles. Among the haemophilia cases recorded in the Universal Data Collection (UDC), 3.47% of neonates experienced bleedings to the CNS connected with birth. The most frequent were subdural bleedings (68.2%), intracerebral haemorrhages (13.6%), bleeding to cerebellar hemispheres (9.0%) and subarachnoid haemorrhages (4.6%) [21].

Similar to other clinical cases described (Tabl. 1), the case presented in this paper had the clinical symptoms and laboratory test results which were characteristic of haemophilia. The first sign of the coagulation disorder was a prolonged bleeding from the injection site which occurred around the 36th hour of the child’s life. Further diagnostics revealed indeterminable APTT, together with normal PT and INR. The bleeding did not stop after the administration of vitamin K and cyclonamine as they do not affect the factor VIII levels, which is why they were ineffective. The examined coagulation factors showed the low level of factor VIII, which confirmed the haemophilia diagnosis.

No IVH was observed during hospitalisation. The effects of coagulation disorders on the presence of bleeding into the CNS are debatable. The analysis of the available clinical cases did not reveal any severe 3rd and 4th degree bleedings to the CNS, even in the case of extreme prematurity (Tabl. 1). It seems that an important role here is rather played by fluctuations in the cerebral blood flow, trauma and the fragile capillary network of the subependymal germinal matrix [22].

Turner syndrome results from sex chromosomes aberration, i.e. from the presence of a single X chromosome. The clinical features of this syndrome include a short stature, sexual infantilism (amenorrhea, infertility) and dysmorphy. The coexisting problems may include heart defects, particularly frequent coarctation of the aorta, or renal defects. Girls with Turner syndrome present a higher risk of type 2 diabetes, Hashimoto’s or coeliac diseases. They can also have problems with vision or recurrent ear infections [23].

More than 60% of patients with Turner syndrome present abnormalities already in the prenatal period, during ultrasound examinations performed in the first trimester [24, 25]. Table 2 presents examples of small dysmorphic features which may occur in the neonatal period in children with Turner syndrome.

**Table 2:** Prenatal and postnatal features of Turner syndrome [23, 26]

Prenatal features	increased nuchal translucency (NT), cystic hygroma, hydrops fetalis, subcutaneous edema, cardiac or renal anomalies, short femur and fetal growth restriction.
Postnatal features	
Physical features	short, webbed neck
	low line of hairs at the back of the neck
	puffy hands, cruses and feet at birth
	lymphedema of the whole body at birth
	Failure to thrive during first year of life
	multiple pigmented naevi
Eyes	epicanthic fold, hiperteloryzm, ptosis
Ears	big, low-set ears rotated to the back, deformity of external ear
Mouth	high arched palate, abnormal dental development, small mandible
Chest	widely spaced nipples, inverted nipples, broad chest (shield chest), pectus excavatum,
Nails	narrow, hyperconvex uplifted nails, nail hypoplasia/dystrophy

The clinical presentation depends on the quantity of the genetic material that was lost [27]. If the entire X chromosome is missing, the symptoms are particularly intensified. The loss of a small chromosomal part can demonstrate no clinical symptoms until adolescence or adulthood. The first symptom may be the primary lack of menstruation or difficulties in getting pregnant.

None of the authors of 8 described clinical cases of haemophilia coexisting with Turner syndrome refers to the clinical signs of Turner syndrome. This may stem from the fact that the majority of the described cases of Turner syndrome are connected with cellular mosaicism, i.e. the presence of two cell lines in one organism, which renders the clinical image of Turner syndrome ambiguous. Another reason may be the fact that dysmorphic features in Turner syndrome are sometimes not immediately noticeable, which makes it more difficult to arrive at this diagnosis.

The coincidence of haemophilia and Turner syndrome is very rare. Only recently has the DNA sequencing provided scientists with the knowledge of the location of specific genes on chromosomes. It has been found that a patient can have two or more genetic diseases, for example, when given genes are located on a single chromosome.

KL Jones et al. described 5 patients with Turner syndrome who were also diagnosed with some other genetic condition, i.e. Li-Fraumeni syndrome, Noonan syndrome, mosaic trisomy 8, pathogenic variant in the RERE gene, and blepharophimosis-ptosis-epicanthanus inversus syndrome [28]. In the course of his clinical medical practice between 2010 and 2017, Jones proved that 1% of 172 patients with TS, who remained under the care of the MGH Turner Syndrome Clinic, had a second genetic disease. Jones also analysed the literature on the subject and evaluated the coincidence of genetic disorders in Turner syndrome. It was found that 51% of patients had autosomal aneuploidy disorders (trisomy 21 being the most frequent) and 24% of the analysed cases had a disease connected with X-linked inheritance [28].

In the event when the primary diagnosis is accompanied by clinical symptoms/dysmorphic features which do not match the image of the said condition, a second coexisting disease should be taken into account.

- Haemophilia and Turner syndrome belong to rare diseases. Their coincidence is extremely rare.
- The first haemophilia symptoms in a preterm child usually occur in the first days of his/her life, which is connected with a significant number of invasive procedures performed at the time.
- Bleeding from an injection site seems to be the most frequent sign of the disease.
- The risk of miscarriage is increased in mother-carriers of the haemophilia gene.
- The risk of massive bleedings into the CNS in preterm children with haemophilia is not increased when compared to healthy preterm infants.
- In a situation when the primary diagnosis is accompanied by clinical symptoms/dysmorphic features which do not match the image of the said condition, a second coexisting disease should be taken into account.

## Data Availability

The datasets analyzed during the current study are available from the corresponding author on reasonable request.

## Abbreviations

APTT: Activated partial thromboplastin time
CNS: Central nervous system
CVS: Chorionic villus sampling
DNA: Deoxyribonucleic acid
ECG: Electrocardiography
FFP: Fresh frozen plasma
INR: International normalized ratio
IVH: Intraventricular hemorrhage
NT: Nuchal translucency
PT: Prothrombin time
rFVIIa: Recombinant factor VIIa
SRY: Sex-determining region Y
TS: Turner syndrome
UDC: Universal Data Collection
vWF: Von Willebrand factor
